# A randomized, controlled, 12-month trial on the effects of an interdisciplinary treatment approach in ESRD patients: study protocol of the smart and fit for kidney transplantation trial (S&F4KTx)

**DOI:** 10.1186/s13063-025-09022-2

**Published:** 2025-08-21

**Authors:** Helge Krusemark, Judith Kleemann, Julian Weigand, Dennis Kannenkeril, Katharina Heller, Tobias Hepp, Marc Albersmeyer, Doris Gerbig, Mario Schiffer

**Affiliations:** 1 Universitätsklinikum Erlangen, Erlangen, Germany; 2 Friedrich-Alexander-Universität Erlangen-Nürnberg, Erlangen, Germany; 3 Fachklinik Bad Heilbrunn, Bad Heilbrunn, Germany

**Keywords:** Prehabilitation, Kidney transplantation, Exercise, Nutrition, Empowerment, Frailty, Body composition, Physical performance

## Abstract

**Background:**

A prolonged waiting time for a kidney transplant leads to a significant decline in the general health status of many patients with chronic kidney disease (CKD). This deterioration may cause these patients to be classified as unsuitable for transplantation during the waiting period owing to frailty, poor body composition, or the development of new comorbidities. Studies have shown that physical exercise and tailored nutrition can maintain and improve physical performance and overall health status.

**Methods:**

This study is a 1:1 controlled, randomized trial. The control group will receive standard care along with an app designed for dialysis patients, which provides information on topics such as nutrition, vital signs, blood parameters, and details about their condition. In addition, the intervention group will receive individualized nutritional and exercise recommendations, along with a 3-week inpatient rehabilitation program. At 0, 6, and 12 months, body composition (body mass index, skeletal muscle mass, body fat mass, and waist circumference) and physical performance (6-min walk test, timed up and go test, sit-to-stand test, one-legged stance test, stair climbing test, and frailty), blood values (e.g., potassium and phosphate), and quality of life (KDQOL) will be evaluated. Bone density (tibia and radius) will be measured at 0 and 12 months.

**Discussion:**

Studies have demonstrated that physical activity and tailored nutrition have positive effects on the general health of dialysis patients. However, pretransplantation preparation programs (prehabilitation) aimed at preserving transplant eligibility have not yet been evaluated. This study aims to address this gap by investigating a potential care program for patients on the transplant waiting list.

**Trial registration:**

ClinicalTrials.gov NCT06040281. Registered on September 15, 2023.

**Trials status:**

Protocol version number: 4 (30.07.2025).

Recruitment start: 18.09.2023.

Recruitment completed: 30.06.2025.

**Supplementary Information:**

The online version contains supplementary material available at 10.1186/s13063-025-09022-2.

## Introduction

### Background

Chronic kidney disease (CKD) presents a significant burden on healthcare systems, with a prevalence of 10% worldwide. Over 2 million people require dialysis due to end-stage kidney disease (ESKD) [1]. Compared with dialysis, kidney transplantation (KT) is widely recognized as the optimal treatment for patients with terminal kidney failure, offering not only improved morbidity but also increased life expectancy and quality of life [2].

In Germany, dialysis patients face an average waiting time of 8–10 years for a kidney transplant [3]. In comparison, the average waiting times in neighboring countries are significantly shorter. In France (which is not part of the Eurotransplant network), the average waiting time ranges between 2 and 3 years [4]. In the Netherlands (which is part of the Eurotransplant network), the average waiting time is similarly between 2 and 3 years [5].

During the waiting period, many patients experience a decline in their overall health status. This deterioration is attributable partly to the dialysis treatment itself and partly to the frequent immobility of patients, who often experience reduced physical capacity under dialysis therapy. Consequently, many patients become ineligible for transplantation or do not survive the waiting period due to cardiovascular disease, which accounts for approximately 46% of all deaths in dialysis patients [6].

In addition, patients eligible for a transplant often exhibit more comorbidities at the time of kidney transplantation, depending on the duration of their waitlisted status. Studies indicate that, regardless of age, more than 50% of transplant recipients are prefrail or frail, which is associated with a 2.2-fold increase in 5-year mortality [7]. The risk of frailty increases with the progression of chronic kidney disease [8], affecting not only older dialysis patients (> 65 years) but also individuals across all age groups [9].

Maintaining a healthy weight is vital for preparing for kidney transplantation. Many patients with chronic renal failure struggle with obesity, which is often accompanied by other cardiovascular risk factors, such as hypertension, insulin resistance, dyslipidemia, atherosclerosis, and metabolic disorders [10]. There is also an increased risk of malnutrition due to dietary restrictions and increased protein requirements.

To reduce these risks, lifestyle modification is essential. Physical activity in combination with nutritional support offers numerous benefits for patients with chronic renal failure, including reduced cardiovascular disease risk through mechanisms such as blood pressure reduction, increased heart rate variability, and increased blood vessel elasticity [11–13]. Increasing skeletal muscle mass through regular physical activity, coupled with improvements in physical performance and optimization of body fat mass, also reduces cardiovascular risk [14]. In addition, physical activity enhances functional capacity, counteracts sarcopenia development, and improves both physical and psychological well-being, thereby increasing patients’ quality of life [15–20]. A large cluster randomized trial (DiaTT; *n* = 1211) has shown that exercise over 12 months can improve physical functionality in people with end-stage renal disease and reduce morbidity as measured by hospitalizations [21].

The nutrition of dialysis patients involves addressing and assessing various individual issues. Given the increasing prevalence of obesity, hypertension, diabetes, and malnutrition, tailored nutrition is essential for optimizing transplantation preparation. This approach can optimize critical blood parameters (potassium, phosphate, protein), prevent sarcopenia, and reduce cardiovascular risk factors [22, 23].

The aim of this study is to investigate whether individualized support in physical activity and nutrition, in combination with a 3-week inpatient rehabilitation program and an informational app, significantly improves body composition and physical performance in patients on the kidney transplant waiting list compared to the use of the informational app alone.

## Materials and methods

### Research hypothesis

Individualized care combined with comprehensive rehabilitation and an informational app is superior to standard care combined with an informational app in terms of body composition and physical performance in patients on the waiting list or pre-waiting list for a kidney transplant.

### Study design overview

The Smart & Fit for Kidney Transplantation trial is designed as a randomized, controlled, monocentric trial with two parallel groups. Patients and care providers will not be blinded as this is not possible due to the intervention design. The study is being conducted at the University Hospital Erlangen in Germany. Recruitment is carried out via the waiting list for kidney transplantation at the Transplantation Center Erlangen-Nuremberg. Patients in the intervention group will undergo 3 weeks of comprehensive rehabilitation in a nephrology rehabilitation clinic or, if they require care and are of a suitable age, in a geriatric rehabilitation clinic. The intervention group will receive individualized exercise and nutritional support for patients with chronic kidney failure, including an informative app about chronic kidney disease, and the control group will receive only the informative app. The patient allocation ratio is 1:1. The outcome measures will be measured at three time points: (1) directly after signing the confirmation of participation, (2) 6 months later, and (3) 12 months later. To ensure comparability between the three measurement time points, the tests are conducted on the same weekday. This is particularly important with regard to dialysis. The SPIRIT reporting guidelines were followed in the preparation of the manuscript [24].

### Study participants

#### Selection criteria

Patients listed on the waiting or pre-waiting list of the Transplantation Center Erlangen-Nuremberg are eligible to participate in this study. Each patient meeting the inclusion criteria will be personally contacted by mail and informed about the study. Additionally, patients registering for an initial consultation at the transplantation center during the trial will be informed about the study. All participants will receive a patient information sheet and will be provided with detailed information about the study and data protection by the study staff on the day of enrollment. It is emphasized that participation is voluntary and they can withdraw their participation at any time without any negative consequences. Informed consent and documents are available in German and English.

Participants must be over 18 years old and already require dialysis. To exclude prior experiences of patients who have already received support, individuals who have previously received personalized guidance regarding exercise and nutrition are not eligible.

During the study period, participation in additional scientific studies is prohibited if these involve a sports- or nutrition-related intervention that could potentially influence the primary or secondary outcomes of the present trial. This restriction is intended to prevent co-intervention bias and to ensure comparability between intervention groups.

Participation in other clinical studies without active sports- or nutrition-related components, as well as the use of medications or treatments as part of routine medical care, remains permitted. Additionally, to access support services, patients must have a mobile device capable of using the app. Patients who are unable to complete the assessments due to psychological or physical limitations are not eligible to participate in the study. This refers exclusively to conditions that, for example, prohibit physical activity. In contrast, patients who rely on assistive devices (e.g., a walker or wheelchair) are eligible to participate.

#### Randomization

After providing informed consent and completing the baseline assessment, the participants will be randomly divided into two groups: (1) an intervention group with individualized exercise and nutritional support, comprehensive rehabilitation, and an informational app or (2) a control group with only the informational app. The randomization of the allocation sequence will be completed via computer-generated random numbers stratified by age (18–30; 31–50; 51–64, and ≥ 65). The statistician responsible will provide the study team with envelopes containing the respective randomization before the appointment, with the envelopes only being opened after completion of the baseline assessment. The study team responsible for implementing the study group assignments does not have access to the allocation schedule and only receives the sealed envelopes. The person responsible for the allocation schedule, on the other hand, has no contact with the participants or the generated data.

### Intervention

#### Control group

All patients will undergo the standard evaluation process for transplantation preparation, which consists of numerous medical assessments designed to minimize the risk of mortality during and after transplantation. These medical examinations include, for example, a pelvic CT scan, ophthalmologic examination, dental examination, colonoscopy, and others. These assessments must be completed before approval for kidney transplantation. Additionally, patients will receive access to a publicly available app that provides information and recommendations regarding kidney disease and nutrition for dialysis patients.

#### Intervention group

The participants in the intervention group will receive access to the same app in addition to standard care. In addition to the standard functions, users can connect the app to the university hospital, allowing vital signs and blood parameters to be displayed directly within the hospital’s system. Participants in the intervention group will also complete a 7-day nutrition and activity log. Personalized nutrition and exercise recommendations are provided on the basis of this log, the results of the baseline assessment, and the blood and vital parameters transmitted through the app. Individualized training plans for exercise, consisting of endurance training guidelines and exercises for resistance and balance training, will be provided to patients. The recommendations are based on the WHO guidelines on physical activity, which include: aerobic exercise for at least 150 min per week at moderate intensity, resistance training twice per week, and balance training twice per week [25]. For nutrition, recommendations tailored to the patient’s goals (weight reduction, weight gain, improvement of blood values, etc.) and easily integrable into daily life will be given.

This individualized care aims to counteract the reduction in physical performance and promote the optimization of body composition. In addition, participants in the intervention group will undergo a 3-week inpatient rehabilitation program, during which they will receive intensive care from physicians, physiotherapists, occupational therapists, sports therapists, psychologists, and nutritionists. The rehabilitation takes place between Visit 2 and Visit 3, as sufficient time must be allocated for the application process and participant preparation. The rehabilitation clinic has confirmed in advance that adequate capacity is available to accommodate participants in the intervention group.

Regular telephone check-ins (every 2 months) will be conducted to monitor and potentially adjust the recommendations between visits. During these telephone check-ins, general well-being is assessed, and adherence to or difficulties with the recommendations are discussed. Study participants are informed about these contacts on the day of enrollment. Regular contact is intended to ensure adherence to the implementation of recommendations as well as compliance with scheduled visits.

### Data collection and outcomes

**Fig. 1:**
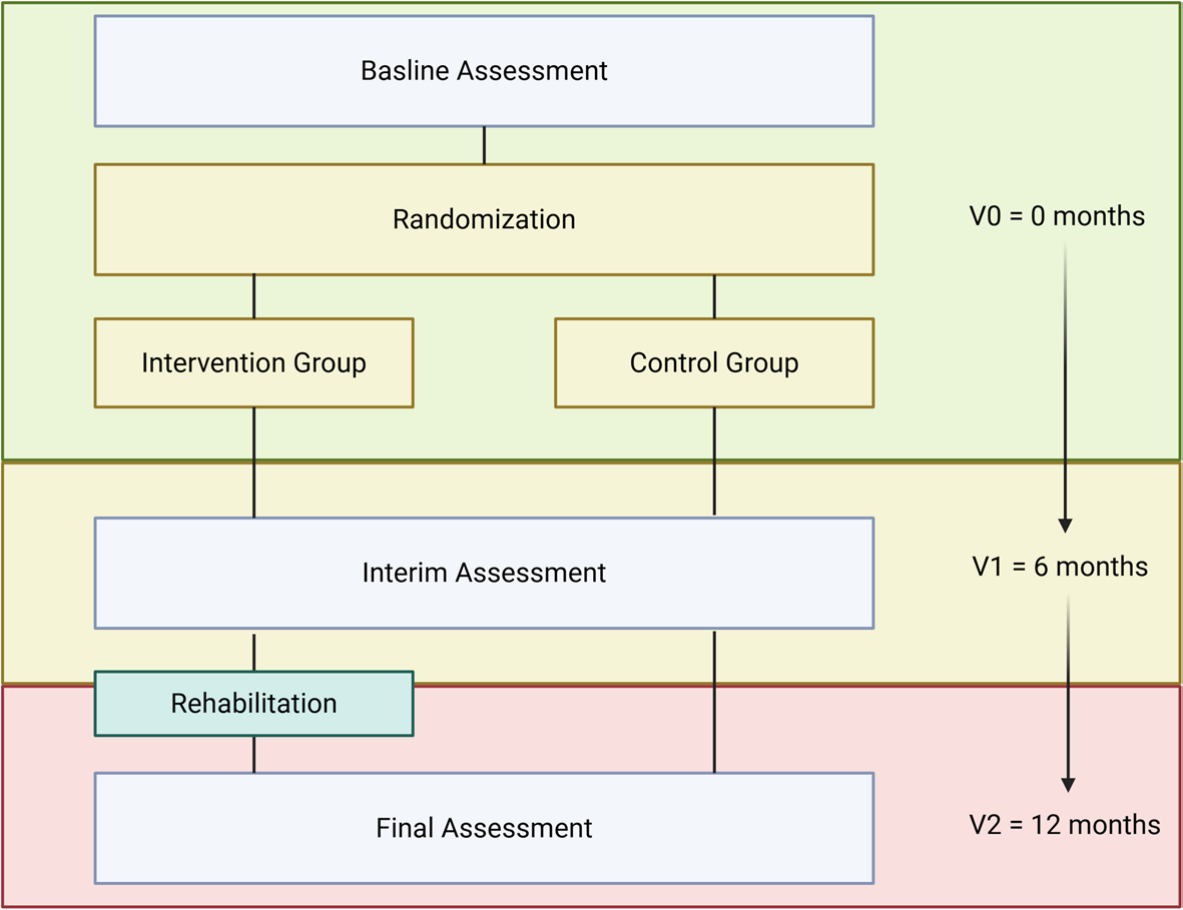
Study timeline (Created in BioRender. Krusemark, H. (2025) https://BioRender.com/f20g408)

The data will be collected according to the study timeline displayed in Fig. 1 and described in Table 1. Each visit follows the same protocol and sequence of examinations, ensuring that there is no bias, particularly with regard to blood values and physical performance outcomes. The data will be entered into an electronic database on the same day and verified by a second study staff member. Only the study team has access to the original data. This electronic database does not contain any personalized data; participants are assigned a study ID, which can only be decoded by the study team. The data can only be accessed through a secure login at the University Hospital Erlangen and are protected by a password. The principal investigator grants access to the de-identified data only to individuals involved in the analysis. A participant will be withdrawn if they undergo transplantation, pass away, or withdraw their informed consent.

**Table 1:** Participant timeline

	Study period			
	Enrollment	Allocation	Post-allocation	
Timepoint	0	0	t_1_	t_2_
Eligibility screen	X			
Informed consent	X			
Allocation		X		
Interventions:				
Intervention group			X	X
Control group			X	X
Assessments:				
Body composition Body mass index Skeletal muscle mass Body fat mass Waist circumference	X		X	X
Physical performance 6-min walking test Sit-to-stand test (60 s) One-legged stance test Timed up and go test Stair climbing test	X		X	X
Frailty	X		X	X
Questionnaires Freiburg Physical Activity Questionnaire Barthel Index KDQOL	X		X	X
Blood parameters	X		X	X
Bone density	X			X

An interim analysis is planned at approximately the midpoint of the overall study duration (after one and a half year). However, this analysis will serve only internal monitoring purposes (e.g., assessment of feasibility, recruitment, and data quality) and is not intended to inform early termination of the study based on efficacy, harm, or futility. A termination of the study based on these results is not anticipated.

The University Hospital Erlangen is, however, obligated to notify the funder if it becomes evident that the research project can no longer be feasibly implemented. In such a case, any decision to discontinue the study prematurely will require prior agreement between the study leadership and the sponsor. No other circumstances could lead to study termination.

Any amendment to the study protocol that may impact the conduct of the study, the potential benefit to the patient, or patient safety, including changes to study objectives, study design, patient population, sample size, study procedures, or significant administrative aspects, requires a formal amendment to the protocol. Such an amendment must first be discussed by the principal investigator with the funder before submission to the ethics committee. Upon approval by the ethics committee, the amendments are then recorded in the trial registries. At this time, no protocol amendments are planned.

Ancillary studies are not planned at this stage. However, participants are asked to provide tiered consent regarding data processing at the time of informed consent. This specifically refers to the potential use of their data for future research without the need for re-consenting. Participants may decline this option and may also withdraw their consent for this purpose at any time. Biological specimens will not be stored in this context.

#### Primary outcomes

In line with KDIGO guidelines recommending the assessment and management of both malnutrition and obesity in transplant candidates, we defined our primary outcome as the absolute deviation from a target BMI of 22.5 kg/m^2^ [26]. This value, corresponding to the midpoint of the normal BMI range (18.5–24.9 kg/m^2^), was selected to reflect the need for weight optimization in both underweight and overweight individuals in order to improve transplant eligibility and outcomes. Globally, BMI cut-off values of 30 or 35 kg/m^2^ are commonly applied as eligibility thresholds for kidney transplantation [27–29]. Therefore, weight adjustment is crucial for affected patients, particularly to reduce the risk of complications during and after transplantation. Malnutrition, however, is also associated with increased perioperative morbidity and mortality. In dialysis patients, malnutrition has been linked to higher mortality rates [30], and in general, it significantly increases the risk of adverse postoperative outcomes [31]. BMI is assessed via bioelectrical impedance analysis (BIA) using the InBody 270 device (Inbody Europe B.V.). This analysis requires height, age, and sex.

#### Secondary outcomes

The secondary outcomes include further alterations in body composition, physical performance, frailty, questionnaires on independence and quality of life, blood parameters, and bone density.

For the assessment of body composition, muscle mass and body fat mass (in kilograms) are additionally measured using bioelectrical impedance analysis, along with waist circumference (in centimeters).

To evaluate physical performance, the 6-min walk test (6MWT) [32], sit-to-stand test (STS60) [33], timed up and go test (TUG) [34], one-legged stance test (OLST) [35], and stair climbing test (SCT) will be conducted. The 6MWT assesses cardiopulmonary endurance by having participants walk a 30-m course for 6 min, covering as much distance as possible. If necessary, participants are allowed to take breaks and resume the test [32]. Lower extremity strength and endurance are evaluated with the STS60. The participants are required to stand up and sit down from a chair without armrests as many times as possible within 60 s. The chair must be securely anchored, and participants must cross their arms over their chest [33]. The TUG test assesses functional mobility. The participants stand up from a chair with armrests, walk to a 3-m mark, turn around, walk back, and sit down again [34]. Balance is evaluated with the OLST. The participant must stand unassisted on one leg, and the duration is timed in seconds from when one foot is lifted off the floor until it touches the ground again. Both sides are tested, with a maximum of 1 min per side [35]. The stair climbing test evaluates functional strength, balance, and agility by asking participants to ascend and descend a specified number of steps. The participants climb up and down one flight of stairs (23 steps) as quickly as possible. They may use the handrail if needed, and this is recorded [36].

Frailty is assessed according to Fried’s criteria, where participants receive a frailty point for each of the following criteria: (1) unintended weight loss in the past year > 4.5 kg, (2) self-reported exhaustion, (3) weakness (low grip strength), (4) slow walking speed, and (5) low physical activity. Participants scoring 1–2 points are considered prefrail, whereas those scoring ≥ 3 points are considered frail. A low handgrip strength is defined as less than 29–32 kg for men and less than 17–21 kg for women, depending on BMI. A slow walking speed is present when it takes more than 6.5–7.7 s, depending on height, to cover a distance of 5 m. Low physical activity is indicated by a weekly energy expenditure of less than 270 kcal for women or 383 kcal for men.

The Freiburg Questionnaire on Physical Activity is used to assess physical activity [37]. Weekly activities are recorded based on their duration and summed accordingly. This tool also allows simultaneous evaluation of adherence to prescribed physical activity. A score of 15 points is required to meet the recommended guidelines.

To assess independence or the need for care, the Barthel Index is used. To evaluate quality of life specifically in dialysis patients, participants will independently complete the Kidney Disease Quality of Life Instrument (KDQOL).

The blood parameters measured in this study include potassium, phosphate, HbA1c, calcium, creatine kinase, albumin, C-reactive protein, ferritin, parathyroid hormone, vitamin D, leukocytes, hemoglobin, and erythrocytes.

Additionally, bone density will be measured at the baseline visit and at the V2 visit via micro-CT. The total, trabecular, and cortical bone mineral densities of the tibia and radius are measured.

In addition to the primary and other secondary endpoints, participants’ status regarding the kidney transplant waiting list will be considered as an additional secondary outcome. Specifically, remaining on the waiting list and successful transplantation will be recorded and analyzed. Participants who are removed from the waiting list will continue to be followed and, whenever possible, will attend follow-up visits so that their data can contribute to the assessment of the intervention’s effectiveness.

### Statistical analysis and power estimates

Primary and secondary outcomes will be analyzed via appropriate bivariate statistical tests to assess significant differences between the study and control groups with respect to the absolute and/or relative differences between measurements at the baseline and at the final examination, i.e., the “differences in the changes.” To account for multiple time points and control the risk of Type I error due to multiple comparisons, a Bonferroni correction will be applied to the significance level.

A more detailed analysis of changes in target variables will be carried out via mixed-effect models, which will also include interim examinations and thus provide a more precise picture of the course of the measurements. Moreover, competing risk analysis can be used to differentiate between the marginal probabilities of different end states, such as transplantation or death. All analyses may be further extended to include the estimation of interaction effects between treatment and patient characteristics such as age, sex, and frailty. As indicated above, several events may occur that end study participation. Successful transplantation as well as death also terminate participation, thereby producing missing values in the follow-up examinations. In the bivariate analyses, these missing values will therefore be replaced with the last value carried forward, as participation was conducted per protocol. For other sources of missing data such as deliberate withdrawal of participation, we can assume a missing-at-random structure and use multiple imputation and the corresponding combining rules to adjust effect estimates. In addition, all analyses involving protocol non-adherent participants will follow the intention-to-treat (ITT) principle, meaning that participants will be analysed according to the group to which they were originally assigned, regardless of their adherence to the intervention. This approach preserves the benefits of randomisation and avoids bias resulting from deviations from the protocol. The ITT approach will not involve imputation to compensate for non-adherence itself, but may be complemented by sensitivity analyses where appropriate. As an alternative, we plan to use a joint model that directly links the longitudinal model to the underlying time-to-event processes to account for the different dropout reasons [38].

The minimum required sample size was determined by calculating the sample sizes needed to reach a statistical power of at least 80% for the main primary outcome, i.e., the body mass index, with a two-sample t test. The required estimate for the standard deviation used in the sample size calculation is estimated from data collected in a previous study, whereas the difference between the mean absolute differences in both groups was specified based on the minimum desired effect size, calculated as the deviation from a reference BMI value of 22 kg/m^2^. This resulted in a required sample size of at least 52 or 70 patients per group to detect a difference of 1 kg/m^2^ with 80% or 90% power, respectively (significance level: 5%, pooled standard deviation: 1.8). To take into account the possible need for multiple test corrections, a final size of at least 168 patients (plus 20%) is aimed for.

### Safety

Since the intervention is not a classic patient-directed intervention but rather a health consultation, there are no intervention-related SAEs or AEs that need to be reported. However, hospitalizations will be recorded during visits and telephone visits. As these hospitalizations cannot be directly related to the intervention, they will not be classified as AEs or SAEs.

## Discussion

The S&F for KTx trial is a large randomized controlled study investigating the role of physical activity and nutritional support during the waiting period for a kidney transplant. It has been scientifically established that cardiovascular risk is the most common cause of death among dialysis patients [39] and that more than half of all patients who receive a kidney transplant are considered frail [7]. Additionally, physical inactivity is particularly pronounced in dialysis patients [40] and represents a significant risk factor for a decline in physical performance and a loss of muscle mass. Although several studies have demonstrated that exercise interventions during dialysis or home exercise programs can optimize body composition and improve physical performance [17, 41–43], widespread implementation has not yet been achieved. This is due to various barriers, including intrinsic factors such as fatigue, frailty, anxiety, or depression, and extrinsic factors such as insufficient support from family or the dialysis center [44].

In terms of nutrition, many patients suffer from either malnutrition [22], particularly protein deficiency, or are significantly overweight [45]. Several studies indicate that an adjusted diet can reduce cardiovascular risk [46, 47] and, in conjunction with physical exercise, can optimize body composition and physical performance [48]. Individual support with regular check-ups is necessary to maintain high adherence. Our study aims to address this gap in support and gather data on whether a centralized point of contact during preparation for transplantation can achieve the positive effects of exercise and optimized nutrition through personalized recommendations and regular monitoring.

Severe obesity leads to a significantly increased risk of wound infections, dehiscence, incisional hernias, delayed graft function, and longer hospital stays after transplantation. Moreover, transplant survival is reduced in individuals with a BMI greater than 35 kg/m^2^ [10]. Conversely, being underweight is also a risk factor for increased mortality. Another factor that predicts poorer outcomes after transplantation is frailty. It increases the length of hospital stay and increases the risk of delayed graft function, mortality, and early hospital readmission [49–51]. Therefore, it is important to optimize body composition and physical performance prior to transplantation.

## Conclusions

In summary, our study aimed to assess the effects of individualized and interdisciplinary care prior to kidney transplantation on physical health. The results will help determine whether such care is beneficial for patients on the waiting list and whether it can optimize body composition and physical performance.

The study results will be published in a peer-reviewed journal article upon completion of data collection, regardless of the outcome. The publication process is intended to be as expedited as possible, with an anticipated duration of 3–4 months following the completion of data collection.

In addition to publication in a journal, the results and interim findings will be presented to professionals at scientific conferences, as well as to the general public at patient information events and on the project website. There are no publication restrictions imposed by the sponsor or other stakeholders.

## Supplementary Information

Supplementary material 1.

## Data Availability

The study team at the University Hospital Erlangen has full access to the datasets. The data will be pseudonymized in a database (RedCap) and made available to the relevant personnel within the University Hospital Erlangen, including the statistician responsible for the analysis.

## References

[1] Liyanage T, Ninomiya T, Jha V, Neal B, Patrice HM, Okpechi I, et al. Worldwide access to treatment for end-stage kidney disease: a systematic review. Lancet. 2015;385(9981):1975–82. 25777665 10.1016/S0140-6736(14)61601-9

[2] Boerstra BA, RB, Astley ME, et al. The ERA registry annual report 2021: a summary. Clin Kidney J. 2023. 10.1093/ckj/sfad281. 38638342 10.1093/ckj/sfad281 PMC11024806

[3] Zecher D, Tieken I, Wadewitz J, Zeman F, Rahmel A, Banas B. Regional differences in waiting times for kidney transplantation in Germany. Dtsch Arztebl Int. 2023;120(23):393–9. 37097064 10.3238/arztebl.m2023.0098 PMC10433364

[4] Bronchard R, Santin G, Legeai C, Bianchi A, Grelier S, Bogue C, et al. Hospital-related determinants of refusal of organ donation in France: a multilevel study. Int J Environ Res Public Health. 2025. 10.3390/ijerph22040618. 40283842 10.3390/ijerph22040618 PMC12026945

[5] Haase-Kromwijk BJ, Heemskerk MB, Weimar W, Berger SP, Hoitsma AJ. Waiting list registration for kidney transplants must improve. Ned Tijdschr Geneeskd. 2017;161: D812. 28378695

[6] Bhandari SK, Zhou H, Shaw SF, Shi J, Tilluckdharry NS, Rhee CM, et al. Causes of death in end-stage kidney disease: comparison between the United States Renal Data System and a large integrated health care system. Am J Nephrol. 2022;53(1):32–40. 35016183 10.1159/000520466

[7] McAdams-DeMarco MA, Law A, King E, Orandi B, Salter M, Gupta N, et al. Frailty and mortality in kidney transplant recipients. Am J Transplant. 2015;15(1):149–54. 25359393 10.1111/ajt.12992 PMC4332809

[8] Roshanravan B, Khatri M, Robinson-Cohen C, Levin G, Patel KV, de Boer IH, et al. A prospective study of frailty in nephrology-referred patients with CKD. Am J Kidney Dis. 2012;60(6):912–21. 22770927 10.1053/j.ajkd.2012.05.017 PMC3491110

[9] Johansen KL, Chertow GM, Jin C, Kutner NG. Significance of frailty among dialysis patients. J Am Soc Nephrol. 2007;18(11):2960–7. 17942958 10.1681/ASN.2007020221

[10] Quero M, Montero N, Rama I, Codina S, Couceiro C, Cruzado JM. Obesity in renal transplantation. Nephron. 2021;145(6):614–23. 33975320 10.1159/000515786

[11] Davies MD, Hughes F, Sandoo A, Alejmi A, Macdonald JH. The effect of exercise on vascular health in chronic kidney disease: a systematic review and meta-analysis of randomized controlled trials. Am J Physiol Renal Physiol. 2023;325(5):F638-55. 37733834 10.1152/ajprenal.00152.2023 PMC10881234

[12] Huang M, Lv A, Wang J, Zhang B, Xu N, Zhai Z, et al. The effect of intradialytic combined exercise on hemodialysis efficiency in end-stage renal disease patients: a randomized-controlled trial. Int Urol Nephrol. 2020;52(5):969–76. 32301053 10.1007/s11255-020-02459-1

[13] Liu X, Hu Z, Xu X, Li Z, Chen Y, Dong J. The associations of plant-based protein intake with all-cause and cardiovascular mortality in patients on peritoneal dialysis. Nutr Metab Cardiovasc Dis. 2020;30(6):967–76. 32249138 10.1016/j.numecd.2020.03.003

[14] Chen CC, Huang YY, Hua Z, Xia L, Li XQ, Long YQ, et al. Impact of resistance exercise on patients with chronic kidney disease. BMC Nephrol. 2024;25(1):115. 38532316 10.1186/s12882-024-03547-5 PMC10967118

[15] Kouidi E. Exercise training in dialysis patients: why, when, and how? Artif Organs. 2002;26(12):1009–13. 12460377 10.1046/j.1525-1594.2002.00937.x

[16] McAdams-DeMarco MA, Ying H, Van Pilsum Rasmussen S, Schrack J, Haugen CE, Chu NM, et al. Prehabilitation prior to kidney transplantation: results from a pilot study. Clin Transplant. 2019;33(1): e13450. 30462375 10.1111/ctr.13450 PMC6342659

[17] Wilkinson TJ, Shur NF, Smith AC. Exercise as medicine in chronic kidney disease. Scand J Med Sci Sports. 2016;26(8):985–8. 27334146 10.1111/sms.12714

[18] Verrelli D, Sharma A, Alexiuk J, Tays Q, Rossum K, Sharma M, et al. Effect of intradialytic exercise on cardiovascular outcomes in maintenance hemodialysis: a systematic review and meta-analysis. Kidney360. 2024;5(3):390–413. 38306116 10.34067/KID.0000000000000361 PMC11000728

[19] Heiwe S, Jacobson SH. Exercise training for adults with chronic kidney disease. Cochrane Database Syst Rev. 2011;2011(10):CD003236. 21975737 10.1002/14651858.CD003236.pub2 PMC10183198

[20] Baiao VM, Cunha VA, Duarte MP, Andrade FP, Ferreira AP, Nobrega OT, et al. Effects of exercise on inflammatory markers in individuals with chronic kidney disease: a systematic review and meta-analysis. Metabolites. 2023. 10.3390/metabo13070795. 37512502 10.3390/metabo13070795 PMC10385645

[21] Anding-Rost K, von Gersdorff G, von Korn P, Ihorst G, Josef A, Kaufmann M, et al. Exercise during Hemodialysis in Patients with Chronic Kidney Failure. NEJM Evid. 2023;2(9):EVIDoa2300057. 10.1056/EVIDoa2300057 38320198

[22] Chan W. Chronic kidney disease and nutrition support. Nutr Clin Pract. 2021;36(2):312–30. 33734473 10.1002/ncp.10658

[23] Perez-Torres A, Caverni-Munoz A, Gonzalez Garcia E. Mediterranean diet and chronic kidney disease (CKD): a practical approach. Nutrients. 2022. 10.3390/nu15010097. 36615755 10.3390/nu15010097 PMC9824533

[24] Chan AW, Tetzlaff JM, Gotzsche PC, Altman DG, Mann H, Berlin JA, et al. SPIRIT 2013 explanation and elaboration: guidance for protocols of clinical trials. BMJ. 2013;346: e7586. 23303884 10.1136/bmj.e7586 PMC3541470

[25] Organisation WH. WHO guidelines on physical activity and sedentary behaviour. Geneva; 2020.

[26] Chadban SJ, Ahn C, Axelrod DA, Foster BJ, Kasiske BL, Kher V, et al. Kdigo clinical practice guideline on the evaluation and management of candidates for kidney transplantation. Transplantation. 2020;104(4S1 Suppl 1):S11–103. 32301874 10.1097/TP.0000000000003136

[27] Chan G, Soucisse M. Survey of Canadian kidney transplant specialists on the management of morbid obesity and the transplant waiting list. Can J Kidney Health Dis. 2016;3:2054358116675344. 28270925 10.1177/2054358116675344 PMC5332083

[28] Dziodzio T, Hillebrandt KH, Knitter S, Nosser M, Globke B, Ritschl PV, et al. Body mass index thresholds and the use of bariatric surgery in the field of kidney transplantation in Germany. Obes Surg. 2022;32(5):1641–8. 35305229 10.1007/s11695-022-06000-4 PMC8986752

[29] Hossain M, Woywodt A, Augustine T, Sharma V. Obesity and listing for renal transplantation: weighing the evidence for a growing problem. Clin Kidney J. 2017;10(5):703–8. 28979783 10.1093/ckj/sfx022 PMC5622900

[30] Rashid I, Sahu G, Tiwari P, Willis C, Asche CV, Bagga TK, et al. Malnutrition as a potential predictor of mortality in chronic kidney disease patients on dialysis: a systematic review and meta-analysis. Clin Nutr. 2024;43(7):1760–9. 38852509 10.1016/j.clnu.2024.05.037

[31] Kondrup J, Allison SP, Elia M, Vellas B, Plauth M, Educational, et al. ESPEN guidelines for nutrition screening 2002. Clin Nutr. 2003;22(4):415–21. 10.1016/s0261-5614(03)00098-0 12880610

[32] Kohl Lde M, Signori LU, Ribeiro RA, Silva AM, Moreira PR, Dipp T, et al. Prognostic value of the six-minute walk test in end-stage renal disease life expectancy: a prospective cohort study. Clinics (Sao Paulo). 2012;67(6):581–6. 22760895 10.6061/clinics/2012(06)06 PMC3370308

[33] Costa MJC, Cavalcanti FCB, Bezerra SD, Araujo Filho JC, Fernandes J, Marinho PEM. Relationship between quadriceps thickness and 60-second sit-to-stand test in patients with chronic kidney disease. J Bras Nefrol. 2022;44(2):164–70. 34519760 10.1590/2175-8239-JBN-2021-0064 PMC9269191

[34] Jung HW, Choi IY, Shin DW, Han K, Yoo JE, Chun S, et al. Association between physical performance and incidence of end-stage renal disease in older adults: a national wide cohort study. BMC Nephrol. 2021;22(1): 85. 33691641 10.1186/s12882-021-02291-4 PMC7945335

[35] Ortega-Perez de Villar L, Martinez-Olmos FJ, Junque-Jimenez A, Amer-Cuenca JJ, Martinez-Gramage J, Mercer T, et al. Test-retest reliability and minimal detectable change scores for the short physical performance battery, one-legged standing test and timed up and go test in patients undergoing hemodialysis. PLoS One. 2018;13(8):e0201035. 10.1371/journal.pone.0201035 PMC6104925 30133445

[36] Dankert A, Neumann-Schirmbeck B, Dohrmann T, Plumer L, Wunsch VA, Sasu PB, et al. Stair-climbing tests or self-reported functional capacity for preoperative pulmonary risk assessment in patients with known or suspected COPD-a prospective observational study. J Clin Med. 2023. 10.3390/jcm12134180. 37445215 10.3390/jcm12134180 PMC10342346

[37] Frey I, Berg A, Grathwohl D, Keul J. Freiburg questionnaire of physical activity–development, evaluation and application. Soz Praventivmed. 1999;44(2):55–64. 10407953 10.1007/BF01667127

[38] Henderson R, Diggle P, Dobson A. Joint modelling of longitudinal measurements and event time data. Biostatistics. 2000;1(4):465–80. 12933568 10.1093/biostatistics/1.4.465

[39] Modi ZJ, Lu Y, Ji N, Kapke A, Selewski DT, Dietrich X, et al. Risk of cardiovascular disease and mortality in young adults with end-stage renal disease: an analysis of the US renal data system. JAMA Cardiol. 2019;4(4):353–62. 30892557 10.1001/jamacardio.2019.0375 PMC6484951

[40] Johansen KL, Chertow GM, Ng AV, Mulligan K, Carey S, Schoenfeld PY, et al. Physical activity levels in patients on hemodialysis and healthy sedentary controls. Kidney Int. 2000;57(6):2564–70. 10844626 10.1046/j.1523-1755.2000.00116.x

[41] Clarkson MJ, Bennett PN, Fraser SF, Warmington SA. Exercise interventions for improving objective physical function in patients with end-stage kidney disease on dialysis: a systematic review and meta-analysis. Am J Physiol Renal Physiol. 2019;316(5):F856–72. 30759022 10.1152/ajprenal.00317.2018

[42] Manfredini F, Mallamaci F, D’Arrigo G, Baggetta R, Bolignano D, Torino C, et al. Exercise in patients on dialysis: a multicenter, randomized clinical trial. J Am Soc Nephrol. 2017;28(4):1259–68. 27909047 10.1681/ASN.2016030378 PMC5373448

[43] Smart NA, Williams AD, Levinger I, Selig S, Howden E, Coombes JS, et al. Exercise & Sports Science Australia (ESSA) position statement on exercise and chronic kidney disease. J Sci Med Sport. 2013;16(5):406–11. 23434075 10.1016/j.jsams.2013.01.005

[44] Delgado C, Johansen KL. Barriers to exercise participation among dialysis patients. Nephrol Dial Transplant. 2012;27(3):1152–7. 21795245 10.1093/ndt/gfr404 PMC3289894

[45] Kramer HJ, Saranathan A, Luke A, Durazo-Arvizu RA, Guichan C, Hou S, et al. Increasing body mass index and obesity in the incident ESRD population. J Am Soc Nephrol. 2006;17(5):1453–9. 16597682 10.1681/ASN.2005111241

[46] Appel LJ, Moore TJ, Obarzanek E, Vollmer WM, Svetkey LP, Sacks FM, DASH Collaborative Research Group, et al. A clinical trial of the effects of dietary patterns on blood pressure. N Engl J Med. 1997;336(16):1117–24. 9099655 10.1056/NEJM199704173361601

[47] Appel LJ, Sacks FM, Carey VJ, Obarzanek E, Swain JF, Miller ER 3rd, et al. Effects of protein, monounsaturated fat, and carbohydrate intake on blood pressure and serum lipids: results of the OmniHeart randomized trial. JAMA. 2005;294(19):2455–64. 16287956 10.1001/jama.294.19.2455

[48] Cheema B, Abas H, Smith B, O’Sullivan A, Chan M, Patwardhan A, et al. Progressive exercise for anabolism in kidney disease (PEAK): a randomized, controlled trial of resistance training during hemodialysis. J Am Soc Nephrol. 2007;18(5):1594–601. 17409306 10.1681/ASN.2006121329

[49] McAdams-DeMarco MA, King EA, Luo X, Haugen C, DiBrito S, Shaffer A, et al. Frailty, length of stay, and mortality in kidney transplant recipients: a national registry and prospective cohort study. Ann Surg. 2017;266(6):1084–90. 27655240 10.1097/SLA.0000000000002025 PMC5360544

[50] Garonzik-Wang JM, Govindan P, Grinnan JW, Liu M, Ali HM, Chakraborty A, et al. Frailty and delayed graft function in kidney transplant recipients. Arch Surg. 2012;147(2):190–3. 22351919 10.1001/archsurg.2011.1229

[51] McAdams-DeMarco MA, Law A, Salter ML, Chow E, Grams M, Walston J, et al. Frailty and early hospital readmission after kidney transplantation. Am J Transplant. 2013;13(8):2091–5. 23731461 10.1111/ajt.12300 PMC4000567

